# Student academic performance in non-lecture physiology topics following the abrupt change from traditional on-site teaching to online teaching during COVID-19 pandemic

**DOI:** 10.1080/10872981.2022.2149292

**Published:** 2022-11-23

**Authors:** Pachara Varachotisate, Natakorn Siritaweechai, Weerapat Kositanurit, Sekh Thanprasertsuk, Maneerat Chayanupatkul, Thana Thongsricome, Thanapob Bumphenkiatikul, Nipat Chuleerarux, Pasakorn Watanatada, Duangporn Werawatganon, Juraiporn Somboonwong, Prasong Siriviriyakul, Sompol Sanguanrungsirikul, Saknan Bongsebandhu-Phubhakdi, Varis Ratanasirisawad, Aunchalee Jaroenlapnopparat, Chuti Burana, Pornpavee Somsirivattana, Onanong Kulaputana, Kasiphak Kaikaew

**Affiliations:** a Department of Physiology, Faculty of Medicine, Chulalongkorn University, Bangkok, Thailand; b Division of Academic Affairs, Faculty of Medicine, Chulalongkorn University, Bangkok, Thailand

**Keywords:** Academic performance, COVID-19, non-lecture based teaching, online learning, physiology, pre-clerkship education, student opinion, undergraduate medical education

## Abstract

**Background:**

During the COVID-19 pandemic, pre-clerkship medical education, including all physiology classes, was obliged to change to online teaching due to limitations of on-site (face-to-face) classes. However, the effectiveness of online teaching in non-lecture physiology topics during the COVID-19 pandemic has not been thoroughly investigated.

**Method:**

We conducted a prospective study to evaluate the students’ academic achievement and opinions on online teaching during the COVID-19 academic year. Academic achievement of 312 students in the COVID-19 year was compared with that of 299 students in the pre-COVID-19 year. Student opinions regarding social interactions and the preferred learning method were also collected.

**Results:**

We found that student academic achievement in the non-lecture physiology topics, assessed by summative scores, was 4.80±0.92 percent higher in the pre-COVID-19 year than in the COVID-19 year (P < 0.01, Cohen’s d = 0.42). Students rated that online classes tended to reduce their interactions with peers and teachers; however, students preferred online learning over traditional on-site learning.

**Conclusions:**

This study pointed out that students’ academic performance related to the physiology topics taught by online non-lecture methods during the COVID-19 pandemic was lower than their performance when the topics were taught by the traditional (on-site) methods, although students reported that they preferred the online teaching. Hence, we suggest that medical teachers should deliberately plan and utilise a variety of tools and techniques when developing online non-lecture classes to preserve the interactivity of the classes, which might overcome this gap in students’ academic performance.

## Background

Teaching medical students during the coronavirus disease 2019 (COVID-19) pandemic has been a challenge for medical schools globally [1]. Thailand encountered the first COVID-19 case in January 2020, and the infection continued to spread to every province by the end of March. In concern of the viral spreading, the Thai government has reacted accordingly with national lockdown, travelling restrictions, physical distancing, and closure of public spaces, including schools and universities [2].

Meanwhile, medical education cannot cease or postpone for a long time. Medical schools worldwide responded by switching from traditional on-site (face-to-face) teaching methods to online teaching methods, particularly for pre-clerkship education, and most of them were reported to have an overall negative impact on undergraduate medical education [3,4] or reported that this required further validation [5]. Medical schools in Thailand must also continue teaching during the lockdown with the restriction of on-site teaching methods, especially in pre-clerkship classes in which basic scientific knowledge rather than clinical experience is emphasised. Traditional on-site physiology teaching in medical schools has a variety of teaching methods, such as lectures, laboratory demonstrations, basic medical skill practical classes, small group discussions, case conferences, and flipped classrooms with student presentations [6,7]. Some medical schools successfully combined on-site and online teaching methods even before the pandemic [8,9]. In 2020, most on-site physiology classes were forced to switch to online classes using various teaching methods, such as recorded/asynchronous lectures (so-called electronic lectures, e-lectures, or e-learning), recorded/asynchronous laboratory demonstrations, live interactive lectures, live small group discussions, and live case conferences; and the outcome of online teaching has been shown to be considerably effective [10–12].

Second-year pre-clerkship medical students from our institution (the Faculty of Medicine, Chulalongkorn University, Bangkok, Thailand) in the year 2020 were in the midst of the transition from on-site to online teaching since they did not encounter the pandemic in their first year. As they experienced both teaching methods (the on-site methods in their first year and the online methods in their second year), it raised our interests and concerns whether their academic achievement was influenced by the switch in teaching methods due to the COVID-19 pandemic. We have reported previously that asynchronous online lecture was not as effective as the traditional on-site lecture for teaching cardiovascular physiology [13]; however, such an effect on non-lecture classes has not been investigated. We also questioned whether online teaching during the COVID-19 pandemic could substitute traditional on-site teaching. Thus, this study was primarily aimed to evaluate the effectiveness of online teaching in non-lecture physiology topics in the second-year pre-clerkship medical students during the COVID-19 pandemic by analysing student academic achievement using their summative scores. In addition, we also explored the students’ opinions on online teaching in the non-lecture physiology topics in the COVID-19 year.

## Methods

### Study population and study design

This study design was a prospective cohort with historical control. We prospectively gathered the data of the second-year medical students in the academic year 2020 (COVID-19 year) and used the data of the second-year medical students from the previous academic year (pre-COVID-19 year) as a control. This is under the assumption that the only difference between these two academic years was the shift from on-site to online teaching, and a similar academic background could be presumed from the similar enrolment criteria of our undergraduate medical program.

**Figure 1.**
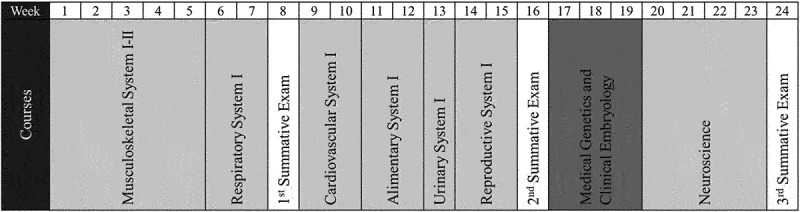
List of the courses taught in the first semester of the second-year medical curriculum.

We aimed to assess the effectiveness of the online non-lecture classes in topics related to physiology in the Faculty of Medicine, Chulalongkorn University, Bangkok, Thailand. This study included 7 physiology topic-containing courses taught in the first semester of the second-year medical curriculum. The list of the courses is shown in Figure 1. Students who enrolled in all the 7 courses and took all summative examinations were included in the study unless they opted out.

Each course was taught by academic staffs from many departments, such as Anatomy, Physiology, and Biochemistry. Summative examinations took place every 7 weeks. It should be noted that the course Medical Genetics and Clinical Embryology was not included in this study since it did not contain a physiology class.

In general, each course aimed to teach basic medical knowledge relevant to the organ system and contained topics of anatomy, physiology, and biochemistry. All topics in each course were designed and approved by the course-coordinating committees, consisting of academic staffs from all relevant departments. Each topic was taught using either lecture or non-lecture methods. Non-lecture methods included laboratory, practicum of physical examination (basic clinical skills), small group discussion, large group discussion, and flipped classroom with student presentation. All physiology topics in the pre-COVID-19 year were taught on-site, whereas all topics in the COVID-19 year were taught online. The transition from on-site to online physiology teaching was planned in a 3-day seminar for all academic staff in the department before the beginning of the semester. To preserve the learning objectives of each class, almost all of the online non-lecture classes were delivered with similar class activities and class materials to those used in the pre-COVID-19 year. Physical examination and clinical investigation classes were taught with live or recorded demonstrations followed by interactive discussions instead of practicums due to limitations in the availability of medical devices and peer physical examination during remote online learning. To preserve the interactivity of traditional on-site non-lecture classes, we organised the online classes using various online interactive tools, such as Zoom meeting with breakout rooms and polls, Kahoot!, Mentimeter, Google Forms, and Google Sheets. All non-lecture physiology topics, duration of the class (in hours), and teaching methods in the pre-COVID-19 year and the COVID-19 year are listed in Table 1.

**Table 1.** List of the non-lecture physiology topics and teaching methods in the pre-COVID-19 year (on-site teaching) and the COVID-19 year (online teaching) and their duration in hours.

Courses	#	Topics	Pre-COVID-19 year (on-site teaching)		COVID-19 year (online teaching)	
			Teaching methods	Duration (hour)	Teaching methods	Duration (hour)
Musculoskeletal System I–II	1	Skeletal muscle	Recorded lab demonstration; Interactive Q&A	1.5	Recorded lab demonstration; Interactive Q&A	1
	2	Isokinetic muscle contraction	Recorded demonstration of lab investigation; Interactive Q&A	1.5	Recorded demonstration of lab investigation; Interactive Q&A	1
	3	Nerve and the neuromuscular junction	Recorded lab demonstration; Interactive Q&A	2	Recorded lab demonstration; Interactive Q&A	2
	4	Reflexes	Practicum	3	Recorded demonstration of physical exam; Interactive Q&A	2
Respiratory System I	5	Physical exam in respiratory system	Practicum	3	Recorded demonstration of physical exam; Interactive Q&A	2
	6	Pulmonary function test	Practicum	2	Recorded demonstration of lab investigation; Interactive Q&A	1
Cardiovascular System I	7	Heart block	Recorded lab demonstration; Interactive discussion	3	Recorded lab demonstration; Interactive discussion	2.5
	8	Electrocardiogram	Practicum	3	Live demonstration of lab investigation; Interactive discussion	2
	9	Heart exam	Practicum	2	Live demonstration of physical exam; Interactive discussion; Recorded wrap-up	2
	10	Blood pressure	Practicum	3	Live demonstration of physical exam; Interactive discussion; Recorded wrap-up	2
	11	Microcirculation	Practicum	3	Live lab demonstration; Interactive discussion; Recorded wrap-up	2
	12	Physical activity and fitness	Practicum	4	Recorded demonstration of lab investigation; Interactive Q&A	2
	13	Cardiovascular disturbances in dogs I	Interactive small group discussion; Interactive wrap-up	3	Interactive small group discussion; Recorded wrap-up	2.5
	14	Cardiovascular disturbances in dogs II	Interactive small group discussion; Interactive wrap-up	3	Interactive small group discussion; Recorded wrap-up	2.5
	15	Cardiovascular disturbances in dogs III	Interactive small group discussion; Interactive wrap-up	3	Interactive small group discussion; Recorded wrap-up	2.5
	16	Case conference in cardiovascular system	Interactive discussion	2	Interactive discussion	2
Alimentary System I	17	Gastrointestinal motility in rats	Live lab demonstration; Interactive discussion	2	Recorded lab demonstration and wrap-up; Interactive Q&A	2
	18	Gastrointestinal secretion in dogs	Recorded lab demonstration; Interactive discussion	3	Recorded lab demonstration and wrap-up; Interactive Q&A	2.5
Urinary System I	19	Renal function in dogs	Recorded lab demonstration; Interactive discussion	3	Recorded lab demonstration; Interactive discussion	3
Reproductive System I	20	Early detection of pregnancy	Practicum; Interactive discussion	3	Recorded lab demonstration; Interactive discussion	3
	21	Gonadal hormones in rats	Live and recorded lab demonstration; Interactive discussion	6	Recorded lab demonstration; Interactive discussion	6
	22	Puberty	Interactive small group discussion; Interactive student presentation	4	Interactive small group discussion; Interactive student presentation	3
	23	Sex education	Interactive small group discussion; Interactive student presentation	4	Interactive small group discussion; Interactive student presentation	3
Neuroscience	24	General and special sensations I (skin, nose, tongue)	Practicum	3	Recorded lab demonstration; Interactive Q&A	3
	25	Special sensation II (eyes)	Practicum	3	Live demonstration of physical exam; Recorded wrap-up; Interactive Q&A	3
	26	Special sensation III (ears)	Practicum	3	Live demonstration of physical exam; Recorded wrap-up; Interactive Q&A	3

Recorded materials were uploaded on the e-learning platforms available for all students, i.e., the MDCU e-learning, myCourseVille, and Echo360, and live online classes (for the COVID-19 year) were taught via Zoom, assisted with online interactive platforms, e.g., breakout rooms and polls of Zoom, Kahoot!, Mentimeter, Google Forms, and Google Sheets.

### Assessment of student academic achievement

Student academic achievement was assessed using summative scores. Summative examinations were scheduled every 7 weeks, as demonstrated in Figure 1. The examinations of both academic years were held on-site by an iPad-based closed-book examination using the application ExamPod (Learning Innovation Center, Chulalongkorn University). The summative examination items of physiology-relevant topics in all courses were 5-option single best answer (SBA). All physiology-relevant examination items were gathered and blindly assessed by 5 medical teachers to determine whether the questions could assess students’ knowledge based on topics taught by non-lecture methods. The items agreed upon by at least 3 out of 5 medical teachers were included in this study. The numbers of included items in each course are listed in Table 2. The summative score of each student, presented as a percentage, represented an individual academic achievement.

**Table 2.** List of physiology classes during the pre-COVID-19 and the COVID-19 years and their numbers of summative items included in the study.

Courses	Duration of the course (week)	Number of non-lecture topics	Duration of online teaching (hour)	Number of included summative items	
				Pre-COVID-19 year	COVID-19 year
Musculoskeletal System I–II	5	4	6	6 (4.8%)	16 (12.2%)
Respiratory System I	2	2	3	10 (7.9%)	6 (4.6%)
Cardiovascular System I	2	10	22	48 (38.1%)	52 (39.7%)
Alimentary System I	2	2	4.5	24 (19.1%)	21 (16.0%)
Urinary System I	1	1	3	15 (11.9%)	11 (8.4%)
Reproductive System I	2	4	15	19 (15.1%)	20 (15.3%)
Neuroscience	4	3	9	4 (3.2%)	5 (3.8%)
Total	18	26	62.5	126 (100%)	131 (100%)

### Item analysis

To confirm the equivalent standard between included summative items of the two academic years, we analysed percent-correct value, discrimination index, and minimal passing level. Percent-correct value (p-value, so-called the difficulty index) of each item was calculated from a proportion of students who chose the correct answer to the total number of responders [14]. We categorised each item into three groups according to the range of the p-value. The items with a p-value of <0.3, 0.3–0.7, and >0.7 were considered difficult, moderate, and easy items, respectively [15].

Discrimination index (DI) was calculated by the difference in the correctness-to-total score ratio of the highest 27^th^ percentile group and the lowest 27^th^ percentile group of the students in each academic year [14]. Ranging from −1 to 1, DI indicates the discrimination power of each item. We categorised items, depending on their discrimination index, into four groups. The items with a DI of <0.15, 0.15–0.24, 0.25–0.34, and >0.35 were considered poor, marginal, good, and excellent items, respectively [15].

Minimal passing level was calculated by the modified Ebel method. All questions were pooled, randomly ordered, and blindly assessed by 7 academic faculties. Each question was assessed for relevance (essential, important, acceptable, or questionable) and easiness (easy, medium, or hard). The expected correct percentage of each question was categorised into a 4 × 3 relevance by easiness table and calculated from an average score from all assessors [16]. An average of the expected correct percentage of all questions indicated the minimal passing level of the examination.

### Class evaluation

At the end of each non-lecture class in the COVID-19 year, a class evaluation form was sent to students using Google Forms comprising two major parts. The first part was the formative assessment which consisted of 10 true/false questions to assess students’ understanding of the topic. The second part of the form was used to assess students’ opinions on whether they achieved the class objectives (a yes/no question), which were informed at the beginning of each class and stated on the first page of the class material. Immediately after submitting the form, students received their formative scores, and the correct answers with explanations of all formative questions were provided. Students were aware that the formative assessment scores did not affect their grades. Student attendance, defined and approved by the course-coordinating committee, in non-lecture classes was recorded by the number of formative evaluation forms submitted within 24 hours after the scheduled date of each class.

To evaluate if better understanding in each non-lecture class could lead to higher academic achievement in the summative exam, we performed a correlation analysis of the formative score with the summative score. The overall formative score was reported as a weighted average score, using the proportion of summative items from each course (the last column in Table 2) as a weighting factor. Students who failed to meet the 80% attendance were not included in the correlation analysis.

### Assessment of student opinions regarding online teaching

At the end of the 7^th^ week (one-third of the semester) and the end of the 23^rd^ week (the end of the semester) in the COVID-19 year, we launched a survey to ask students’ opinions regarding online teaching. Students anonymously and voluntarily rated the level of their interaction with peers and with teachers (lower/similar/higher) when learning non-lecture classes by online methods compared with the on-site methods they had experienced in the previous academic year (before the COVID-19 pandemic). In addition, students were asked if they preferred learning non-lecture topics by online methods compared with the on-site methods they had experienced in the previous academic year. Content validity of the survey was determined by 5 independent experts. The index of item-objective congruence was 0.93, indicating an acceptable quality of the survey [17].

### Ethical considerations

This study was approved by the Institutional Review Board of the Faculty of Medicine, Chulalongkorn University, Bangkok, Thailand (COA no. 900/2020), and the study protocol was carried out in compliance with the international guidelines for human research protection as the Declaration of Helsinki, the Belmont Report, and the CIOMS Guideline.

### Statistical analysis

Statistical analyses were performed using Microsoft Excel (Version 2111), GraphPad Prism (version 9.3.1), and IBM SPSS (version 29.0.0.0). Categorical data were shown as frequency and percentage. Continuous data were shown as mean and standard deviation (SD). The differential distributions of p-value, DI, and student opinions on interactions and the preferred learning method between the 7^th^ and 23^rd^ weeks were analysed using Chi-square test. The difference in summative scores and minimal passing levels between the two academic years were analysed using unpaired *t* test. The standardised effect size (Cohen’s *d*) was calculated as the mean difference divided by the pooled SD. In addition, the difference in summative scores adjusted for possible covariates, i.e., baseline characteristics of students, between the two academic years were analysed with ANCOVA. The correlation between the weighted average formative scores and summative scores was analysed using Pearson correlation. A *P* value of <0.05 was considered statistically significant.

## Results

**Figure 2.**
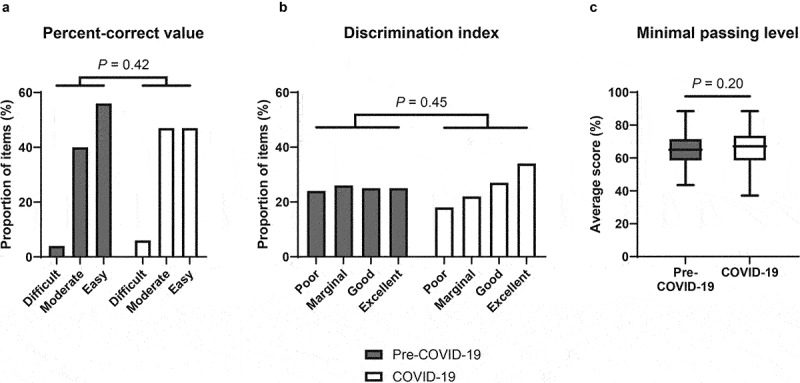
Item analysis.

**Figure 3.**
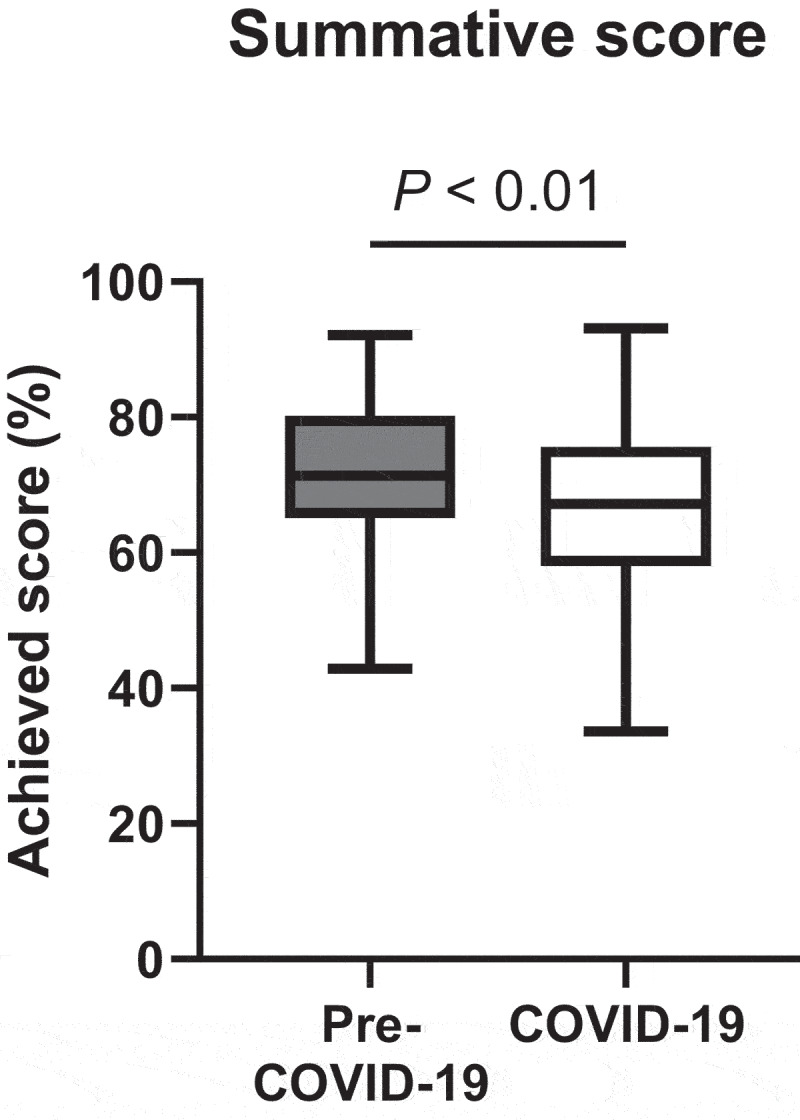
Student academic performance.

There were 299 and 312 second-year medical students in the pre-COVID-19 year and the COVID-19 year enrolled in the study, respectively. Both groups showed no difference in baseline characteristics, including the baseline academic performance assessed by average summative scores of the two courses that contained physiology topics and were taught by the same on-site methods in their first-year curriculum (Table 3). Our medical faculties agreed to include a total of 126 summative items from the pre-COVID-19 year and 131 items from the COVID-19 year. The quality of the summative examinations between the two academic years was equivalent in terms of the percent-correct value and the discrimination index shown in Figure 2(a,b). Also, the minimal passing level calculated by the modified Ebel method did not significantly differ between the two academic years (65.37 ± 9.21% in the pre-COVID-19 year vs. 66.05 ± 10.10% in the COVID-19 year, *P* = 0.20, Figure 2(c). Student academic achievement in the non-lecture physiology topics assessed by summative scores was 4.80 ± 0.92% higher in the pre-COVID-19 year compared to scores in the COVID-19 year (71.23 ± 10.46% vs. 66.42 ± 12.13%, *P* < 0.01, Cohen’s *d* = 0.42, Figure 3). Of note, the difference in student academic achievement between the two academic years remained significant after being adjusted for baseline characteristics (mean difference 4.99 ± 0.58%, *P* < 0.01).

**Table 3.** Baseline characteristics of the students enrolled in the study.

Student characteristics	Pre-COVID-19 year (n = 299)	COVID-19 year(n = 312)	*P* value
Female sex	137 (46%)	160 (51%)	0.18
Age (year)	19.53 ± 0.67	19.60 ± 1.13	0.35
Credits registered before enrolling in the study	43.28 ± 1.07	43.49 ± 2.11	0.13
Credits achieved before enrolling in the study	43.03 ± 0.52	43.00 ± 0.00	0.31
Credits registered in the semester of the study	21.00 ± 0.00	21.00 ± 0.00	1.00
Summative scores in the previous academic year (%)*	82.31 ± 11.92	83.33 ± 12.11	0.30

Categorical data are presented as the number (percentage). Continuous data are presented as mean ± SD. *Summative scores were calculated from the courses that contained physiology classes, i.e., Fundamentals of Tissue Biology and Human Function, and Endocrine System I.

Graphs present a) percent-correct value, b) discrimination index, and c) minimal passing level of each item of the summative examinations in the pre-COVID-19 year and the COVID-19 year. The minimal passing level is presented by a box-and-whisker plot with the Tukey method [boxes indicating an interquartile range (IQR), whiskers indicating 1.5×IQR, and lines in the box indicating the median].

The students’ academic performance or the summative score is presented by a box-and-whisker plot with the Tukey method [boxes indicating an interquartile range (IQR), whiskers indicating 1.5×IQR, and lines in the box indicating the median].

In the COVID-19 year, 303 out of 312 students (97%) attended at least 80% of all non-lecture physiology classes. An average of formative scores, the number of class attendance, and the number of students stating that they achieved the class objectives for each topic are shown in Table 4. The overall weighted average of the formative score was equal to 78.19 ± 9.22%. Pearson correlation analysis between the formative and summative scores was weakly positively correlated (r = 0.29, *p* < 0.01, graph not shown).

**Table 4.** Formative score, class attendance, and class objective achievement of each non-lecture physiology topic.

Courses	#	Topics	Formative score	Class attendance	Class objective achievement
Musculoskeletal System I–II	1	Skeletal muscle	7.52 ± 1.85	303 (97.12%)	283 (93.40%)
	2	Isokinetic muscle contraction	8.01 ± 1.56	300 (96.15%)	282 (94.00%)
	3	Nerve and the neuromuscular junction	8.05 ± 1.78	307 (98.40%)	275 (92.91%)
	4	Reflexes	7.21 ± 2.14	306 (98.08%)	279 (93.94%)
Respiratory System I	5	Physical exam in respiratory system	8.45 ± 1.70	292 (93.59%)	264 (93.29%)
	6	Pulmonary function test	7.23 ± 2.16	291 (93.27%)	257 (91.46%)
Cardiovascular System I	7	Heart block	7.98 ± 1.96	312 (100%)	273 (89.80%)
	8	Electrocardiogram	8.14 ± 1.82	310 (99.36%)	275 (91.67%)
	9	Heart exam	7.83 ± 1.87	308 (98.72%)	278 (93.92%)
	10	Blood pressure	6.66 ± 1.99	309 (99.04%)	283 (96.59%)
	11	Microcirculation	6.79 ± 2.28	308 (98.72%)	262 (91.29%)
	12	Physical activity and fitness	7.29 ± 1.87	308 (98.72%)	265 (92.01%)
	13	Cardiovascular disturbances in dogs I	7.54 ± 1.85	312 (100%)	276 (92.62%)
	14	Cardiovascular disturbances in dogs II	7.59 ± 1.85	311 (99.68%)	282 (95.27%)
	15	Cardiovascular disturbances in dogs III	8.16 ± 1.58	311 (99.68%)	283 (94.65%)
	16	Case conference in cardiovascular system	8.40 ± 2.01	287 (91.99%)	260 (93.53%)
Alimentary System I	17	Gastrointestinal motility in rats	8.35 ± 1.86	236 (75.64%)	195 (91.12%)
	18	Gastrointestinal secretion in dogs	7.31 ± 2.39	285 (91.35%)	255 (95.86%)
Urinary System I	19	Renal function in dogs	7.72 ± 1.81	309 (99.04%)	277 (89.64%)
Reproductive System I	20	Early detection of pregnancy	8.30 ± 1.51	303 (97.12%)	293 (96.70%)
	21	Gonadal hormones in rats	7.48 ± 1.85	309 (99.04%)	289 (93.53%)
	22	Puberty	8.39 ± 1.72	301 (96.47%)	291 (93.87%)
	23	Sex education	7.79 ± 1.78	296 (94.87%)	288 (95.68%)
Neuroscience	24	General and special sensations I (skin, nose, tongue)	7.60 ± 2.10	238 (76.28%)	219 (97.33%)
	25	Special sensation II (eyes)	8.03 ± 1.50	230 (73.72%)	221 (98.22%)
	26	Special sensation III (ears)	6.60 ± 1.75	199 (63.78%)	187 (96.89%)

The formative score is presented as mean ± SD (each topic has a maximum score of 10). Class attendance and class objective achievement are presented as the number of responders (percentage). The class objective achievement was collected by utilising students’ self-assessment form at the end of the class and calculated as a percentage of students who voluntarily answered this question.

Students’ opinions on interactions with peers and with teachers and their preference between online and on-site methods at the 7^th^ and 23^rd^ weeks are shown in Table 5. In general, students reported that online classes resulted in a lower or similar level of interaction with peers, but a similar level of interaction with teachers, compared to the on-site teaching methods. It should be noted that the patterns of responses in the 23^rd^ week (the end of the semester) were similar to the patterns in the 7^th^ week. However, the distributions of answers towards lower levels of student-student and student-teacher interactions were potentiated in the 23^rd^ week (*P* < 0.01, Table 5). Interestingly, students reported that they preferred online learning to on-site learning, although they reported lower levels of interactions.

**Table 5.** Student opinions on the interactions and learning method preference.

Level of interaction with peers	Lower	Similar	Higher	*P* value
week 7 (n = 174)	64 (36.78%)	76 (43.68%)	34 (19.54%)	<0.01
week 23 (n = 265)	144 (54.34%)	108 (40.75%)	13 (4.91%)	
**Level of interaction with teachers**	**Lower**	**Similar**	**Higher**	
week 7 (n = 174)	40 (22.99%)	107 (61.49%)	27 (15.52%)	<0.01
week 23 (n = 264)	88 (33.33%)	156 (59.09%)	20 (7.58%)	
**Learning method preference**	**On-site**	**No preference**	**Online**	
week 7 (n = 173)	48 (27.75%)	48 (27.75%)	77 (44.51%)	0.91
week 23 (n = 266)	74 (27.82%)	69 (25.94%)	123 (46.24%)	

Students chose whether online learning resulted in lower/similar/higher levels of interaction with other students (interaction with peers) and interaction with teachers compared to traditional on-site learning. Students rated if they preferred on-site learning, online learning, or no preference for either method. Data are shown as the number (percentage).

## Discussion

Teaching physiology during the COVID-19 pandemic has been challenging due to the restrictions that limit face-to-face interactions between teachers and students [5]. In this study, we found that the effectiveness of teaching non-lecture physiology topics by online methods was apparently lower than teaching by on-site methods, as indicated by the lower overall academic performance in the COVID-19 year. This finding confirmed the concern of many medical teachers that online learning methods would interfere with and limit students’ academic achievement [18].

Although various online interactive tools and teaching methods were utilised to preserve social interactions in learning experiences during online classes, students reported decreased levels of interactions between them and peers or teachers. It was reported that student interaction in an online class was a determinant of student perceived learning outcome and satisfaction [19]. However, we cannot conclude the causal relationship between students’ perceived interaction and their academic achievement by this study design due to the anonymity of questionnaire responses. Interestingly, we found that while students admitted decreased levels of interactions during online classes, most of the students still preferred online learning. We presumed that this could be because of the benefits of online learning, i.e., self-scheduled learning, repeatability, and travel-free routine [20]. Accordingly, teachers could aim to improve student interaction in online classes through various strategies, such as using flipped classrooms, breakout online conference rooms, and encouragement techniques which will ultimately improve students’ soft skills [21–23].

Formative assessment with prompt feedback has generally been used in medical education. In the COVID-19 year, we used online formative assessment to help teachers assess students’ attendance and class objective achievement. As expected, we found that formative assessments could partly reflect students’ academic performance since the formative score showed a weakly positive correlation with the summative score. This finding added to its well-known benefits demonstrated in many previous studies that online formative assessment could improve students’ understanding, engagement, and self-awareness of the learning outcomes [24–26]. However, developing online formative assessment with online teaching to maximise student learning success is still challenging.

Assessment of student academic achievement has also been influenced by the pandemic, leading to a rapid increase in the implementation of technology-enhanced assessment [27]. For example, the examination during the COVID-19 academic year was shifted from an on-site closed-book examination to an online open-book examination [10] or an online platform with remote invigilation [4,28]. Since it might be inaccurate to directly compare the students’ academic achievement from different modes of examination, we used the same mode of examination (on-site closed-book examination) in our study to avoid this disparity between the two academic years.

To our knowledge, this was the first prospective study that was designed prior to the beginning of the COVID-19 semester; evaluated the academic achievement of students from the two academic years with identical baseline characteristics; and compared the academic achievement in the nearly identical curriculum structure, course-coordinating committees, and mode of summative examination. Moreover, this study contained a large sample size as we collected data from an entire class of students from one of the biggest medical schools in Thailand.

A limitation of this study is that assessment of student knowledge is not the only aspect to be evaluated to fully assess the success of online teaching during the COVID-19 pandemic. Laboratory skills, clinical skills, and other soft skills were not included in this study since they could not be fully assessed by summative scores. Students’ academic achievement by online teaching in other subjects, e.g., anatomy, biochemistry, pharmacology, and pathology, might differ from this study due to a difference in the nature of the subjects [29]. Other factors which were not investigated in this study could possibly affect students’ learning outcomes, so our finding of lower academic performance during the COVID-19 year might not solely be attributable to changing from on-site to online teaching. Since the COVID-19 pandemic suddenly shifted our pre-clerkship classes to online teaching, the academic staff might have insufficient time or inadequate experience in organising online classes, particularly for a large group of students like in our medical program, which might be the cause of the diminished academic performance. In addition, stress levels among medical students have been reported to be another important factor contributing to students’ academic performance [30,31]. However, since our study did not evaluate levels of stress and anxiety among medical students and teachers during the COVID-19 pandemic, we cannot exclude the direct effects of stress and anxiety on students’ academic performance. Hence, further studies are required to evaluate the overall effects of the pandemic on medical students’ academic performance.

## Conclusion

In terms of the academic performance of medical students, online teaching for non-lecture physiology topics during the COVID-19 pandemic appeared to be less effective than the traditional (on-site) teaching methods. Although there are various advanced technologies to assist online learning for pre-clerkship medical courses, it could not completely substitute traditional on-site teaching. Moreover, since students reported a preference for online learning over on-site learning, we suggest that medical teachers should deliberately plan and utilise a variety of tools and techniques when developing online non-lecture classes to preserve the interactivity of the classes, which might overcome this gap in the academic performance of students. However, further research is still required to fully demonstrate the contributing factors to the lower academic performance influenced by online teaching.
